# Unmanned Aerial Vehicle (UAV) Robot Microwave Imaging Based on Multi-Path Scattering Model

**DOI:** 10.3390/s22228736

**Published:** 2022-11-11

**Authors:** Zhihua Chen, Xinya Qiao, Pei Wu, Tiancai Zhang, Tao Hong, Linquan Fang

**Affiliations:** 1 Southwest Technology and Engineering Research Institute, Chongqing 400039, China; 2 Beijing Institute of Technology, Beijing 100081, China; 3 School of Electronics and Information Engineering, Beihang University, Beijing 100191, China; 4 Yunnan Innovation Institute·BUAA, Kunming 650233, China

**Keywords:** UAV robot microwave imaging, multi-path scattering model, UAV networking using satellite transmitting signals

## Abstract

Unmanned Aerial Vehicle (UAV) robot microwave imaging systems have attracted comprehensive attention. Compared with visible light and infrared imaging systems, microwave imaging is not susceptible to weather. Active microwave imaging systems have been realized in UAV robots. However, the scattering signals of geographical objects from satellite transmitting systems received by UAV robots to process imaging is studied rarely, which reduces the need of load weight for the UAV robot. In this paper, a multi-path scattering model of vegetation on the earth surface is proposed, and then the microwave imaging algorithm is introduced to reconstruct the images from the UAV robot receiving the scattering data based on the multi-path model. In image processing, it is assumed that the orbit altitude of a transmitter loaded on the satellite remains unchanged, and the receiver loaded UAV robot obtains the reflective information from ground vegetation with different zenith angles. The imaging results show that the angle change has an impact on the imaging resolution. The combination of electromagnetic scattering model and image processing method contributes to understanding the image results and the multi-path scattering mechanisms of vegetation, which provide a reference for the research and development of microwave imaging systems of UAV robot networking using satellite transmitting signals.

## 1. Introduction

Unmanned Aerial Vehicles (UAVs) are also known as flying robots [1]. The vision system of a UAV robot has a computer-controlled imaging device that allows the robot to see and adjust its flight path accordingly, which helps the UAV robot perceive, fly, avoid obstacles, and to be successfully used for search and rescue [2]. However, the vision system of a UAV robot based on visible light and infrared are sometimes greatly affected by haze, cloud and rain. Microwaves can observe day and night, and are not affected by weather and some buildings. If the UAV robot is equipped with an active microwave imaging system, the total load will be increased. In this study, we will mainly consider UAV robot microwave imaging using the satellite transmitting signals to the target and the ground, and then scattering to the UAV sensors. If the UAV robot just receives the microwave signals to process imaging, which is not equipped with a microwave transmitting source, it will reduce the need of additional load weight for the UAV robot.

A satellite communication system can be used in UAV networking [3]. In addition, the signals transmitted by satellite communication systems receiving by UAV can not only be used in wireless transmissions, but also in processed imaging. Recently, the active radar imaging method of UAV has been researched comprehensively [4]. However, the passive imaging, not equipped with a microwave transmitting source, using multi-path scattering of ground received by UAV robot based on the satellite communication systems is rarely studied.

Furthermore, theoretical studies have shown that the use of a second receiver in addition to a conventional monostatic system, i.e., a transmitter–receiver, forming a multistatic system, could improve the retrieval performance of vegetation and soil biogeophysical variables [5,6]. The passive imaging using multi-path scattering from the ground of a UAV robot can be considered as a multistatic system. In addition, the passive imaging using multi-path scattering of ground received by UAV robot based on the satellite communication systems possesses advantages such as collecting additional information of the target, more flexibility and higher imaging resolution compared to UAV robot active radar imaging because of its separated transmitter and receiver on different platforms [7,8,9,10,11].

To assess the performance of bistatic SAR acquisitions for crops, L. Guerriero [12] presented a theoretical study of the microwave remote sensing of vegetated surfaces. Although the sensitivity study of the observation angle was explored preliminarily based on bistatic measurements, no conclusion about how different observation angles affect the imaging results was drawn. In order to study the bistatic scattering mechanism from the ground, S. Bellez [13] developed a volume-electric-field-integral equation based on a method of moments to simulate bistatic radar scattering from the different sets of cylinders, and then undertook an experiment to verify it. Lastly, a near-field reconstruction algorithm was applied to generate images based on both theoretical and experimental scattered fields. However, since the model is from numerical solution of volume integral equation, the research object is limited to a simple reduced-scale model composed of dielectric parallelepipeds, and thus the image results don’t reflect the relatively complex environment and spatial structure of vegetation appropriately. In this paper, to study the passive imaging using multi-path scattering from ground of UAV robot based on the satellite communication systems, a multi-path scattering scattering model is developed to simulate a scattering field from the virtual three-dimensional structure of vegetation, and then scattering fields of vegetation at different frequency points and positions are processed to obtain the passive multi-path scattering images.

This paper is organized as follows. A multi-path scattering model for vegetation is developed in Section 2. Section 3 introduces the passive image processing algorithm using multi-path scattering from the ground of a UAV robot. In Section 4, simulation results are presented and reasonable analysis is given. Conclusions are drawn in Section 5.

## 2. Model Description

### 2.1. Multi-Path Scattering Model

In this study, the multi-path scattering model for virtual vegetation is established based on Distorted Born Approximation (DBA) [14]. The virtual vegetation is composed of dielectric cylinders and disks which stand for the trunk, branches and leaves, respectively. The ground surface, which is described by the standard deviation of the height, correlation length and dielectric constant, is divided into many small units equally. The total scattering fields are added coherently from all the scatterers from vegetation and ground surface.

**Figure 1:**
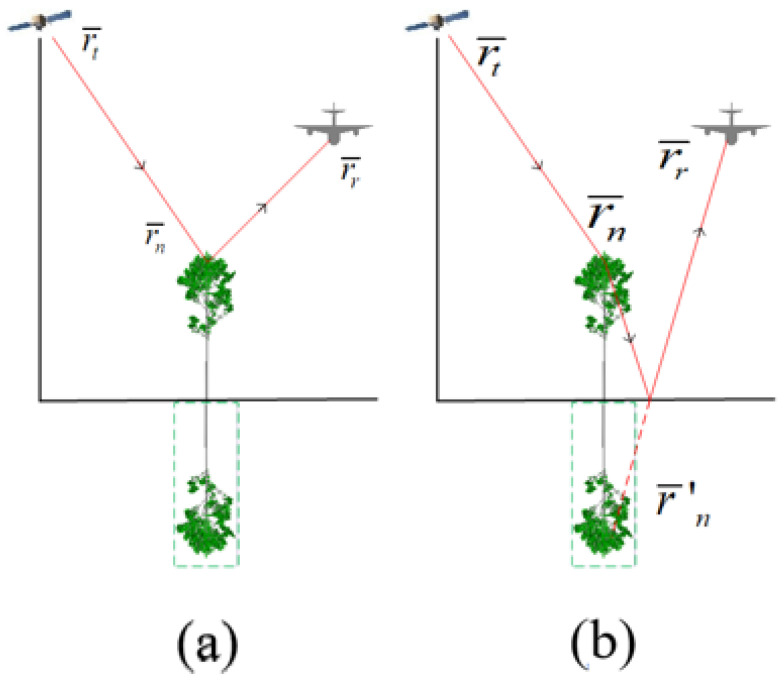

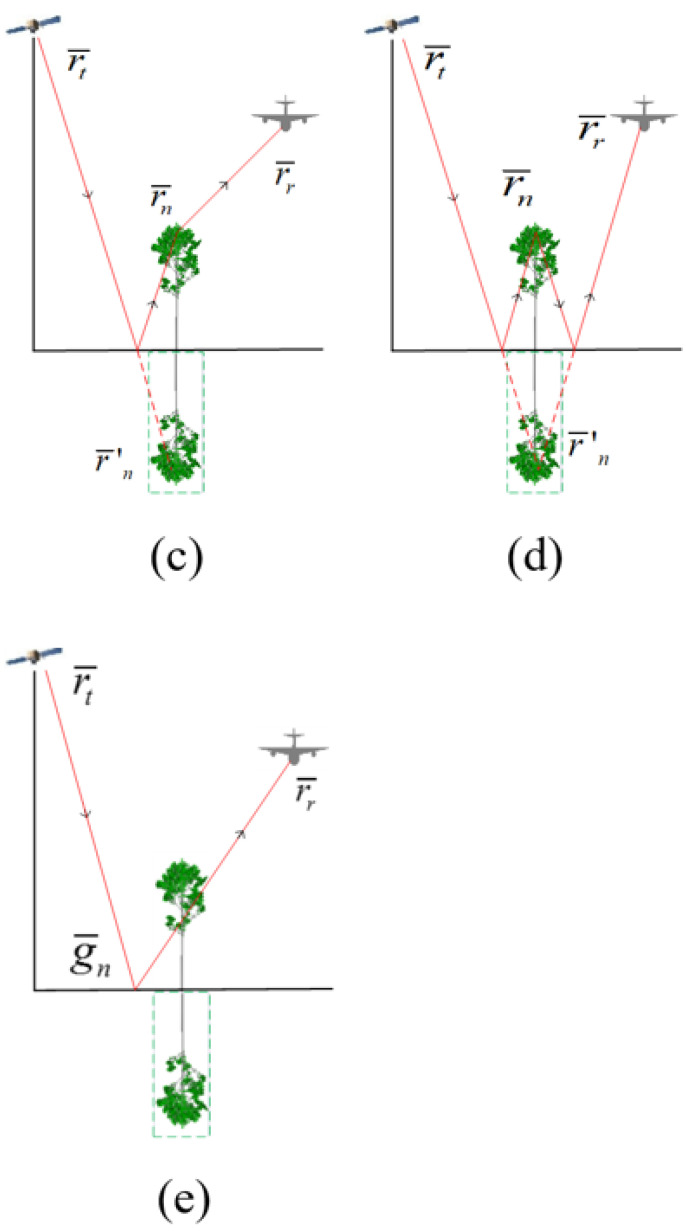
The five scattering components: (**a**) Direct scattering from scatterer; (**b**) Single bounce from scatterer to ground; (**c**) Single bounce from ground to scatter; (**d**) Double bounce from ground, scatter to ground; (**e**) Direct scattering from ground.

It is assumed that there are *N* scatterers in the vegetation area and *M* ground blocks. The total scattering fields include five components, in which four components are about vegetation scatterers with transmission paths inherited from Born approximation and one component is related to ground surface, as shown in Figure 1. Direct scattering from the scatterers in the vegetation is denoted as ${\bar{E}}_{n}^{ds}\left(k_{i},x\right)$. The single bounce of the scattering with a path of transmitter-scatterer-ground-receiver is denoted as ${\bar{E}}_{n}^{sg}\left(k_{i},x\right)$, and the scattering with a path of transmitter-ground-scatterer-receiver is denoted as ${\bar{E}}_{n}^{gs}\left(k_{i},x\right)$. Furthermore, the double bounce of the scattering with a path of transmitter-ground-scatterer-ground-receiver is denoted as ${\bar{E}}_{n}^{gsg}\left(k_{i},x\right)$. Lastly, direct scattering from the ground is denoted as ${\bar{E}}_{m}^{dg}\left(k_{i},x\right)$. Therefore, the total scattering field is the coherent summation of each component from all the azimuth observation positions *x* and wave numbers $k_{i}$, which can be expressed as

(1) $$\bar{E}\left(k_{i},x\right)=\sum_{n=1}^{N}\left({\bar{E}}_{n}^{ds}\left(k_{i},x\right)+{\bar{E}}_{n}^{sg}\left(k_{i},x\right)+{\bar{E}}_{n}^{gs}\left(k_{i},x\right)+{\bar{E}}_{n}^{gsg}\left(k_{i},x\right)\right)+\sum_{m=1}^{M}{\bar{E}}_{m}^{dg}\left(k_{i},x\right)$$

where *n* denotes the scatterer in the vegetation area, and m denotes the ground block. Each component can then be expressed as

(2) $${\bar{E}}_{n}^{ds}\left(k_{i},x\right)=\frac{e^{ikr}}{4\pi r}e^{i\varphi_{n}}A_{t}\left(\theta_{i},\phi_{i}\right)A_{r}\left(\theta_{r},\phi_{r}\right)S_{n}^{ds}{\bar{E}}_{0}^{i}$$

(3) $${\bar{E}}_{n}^{sg}\left(k_{i},x\right)=\frac{e^{ikr}}{4\pi r}e^{i\varphi_{n}}A_{t}\left(\theta_{i},\phi_{i}\right)A_{r}\left(\theta_{r},\phi_{r}\right)S_{n}^{sg}{\bar{E}}_{0}^{i}$$

(4) $${\bar{E}}_{n}^{gs}\left(k_{i},x\right)=\frac{e^{ikr}}{4\pi r}e^{i\varphi_{n}}A_{t}\left(\theta_{i},\phi_{i}\right)A_{r}\left(\theta_{r},\phi_{r}\right)S_{n}^{gs}{\bar{E}}_{0}^{i}$$

(5) $${\bar{E}}_{n}^{gsg}\left(k_{i},x\right)=\frac{e^{ikr}}{4\pi r}e^{i\varphi_{n}}A_{t}\left(\theta_{i},\phi_{i}\right)A_{r}\left(\theta_{r},\phi_{r}\right)S_{n}^{gsg}{\bar{E}}_{0}^{i}$$

(6) $${\bar{E}}_{n}^{dg}\left(k_{i},x\right)=\frac{e^{ikr}}{4\pi r}e^{i\varphi_{n}}A_{t}\left(\theta_{i},\phi_{i}\right)A_{r}\left(\theta_{r},\phi_{r}\right)S_{n}^{dg}{\bar{E}}_{0}^{i}$$

where $A_{t}\left(\theta_{i},\phi_{i}\right)$ and $A_{r}\left(\theta_{r},\phi_{r}\right)$ denote the antenna patterns of the transmitter and receiver, respectively. $\left(\theta_{i},\phi_{i}\right)$ and $\left(\theta_{r},\phi_{r}\right)$ are the transmitting and receiving angles. $\varphi_{n}$ is phase compensation term of the $n_{th}$ scatterer, which is the phase shift from the local to the global coordinate system. Here $\varphi_{n}=({\bar{k}}_{i}-{\bar{k}}_{s})\cdot {\bar{r}}_{n}$, where ${\bar{r}}_{n}$ denotes position vector of the $n_{th}$ scatterer in global coordinate system. ${\bar{k}}_{i}$ and ${\bar{k}}_{s}$ are the wave vectors of incidence and scattering wave, respectively. In addition, $\varphi_{m}=({\bar{k}}_{i}-{\bar{k}}_{s})\cdot {\bar{r}}_{m}$, where ${\bar{r}}_{m}$ denotes the position vector of the $m_{th}$ ground block. The expression of $S_{n}^{ds}$, $S_{n}^{sg}$, $S_{n}^{\mathrm{gs}}$, $S_{n}^{gsg}$ and $S_{n}^{\mathrm{dg}}$ can be written as

(7) $$S_{n}^{ds}=\overset{=\;}{T_{n}^{i}}\overset{=\;}{F_{n}^{s}}\left(\theta_{i},\phi_{i};\theta_{r},\phi_{r},k_{i}\right)\overset{=\;}{T_{n}^{ir}}$$

(8) $$S_{n}^{sg}=e^{i\varphi_{n1}}\overset{=\;}{T_{n}^{i}}\overset{=\;}{F_{n}^{s}}\left(\theta_{i},\phi_{i};\pi -\theta_{r},\phi_{r},k_{i}\right)\overset{=\;}{T_{n}^{rr}}\overset{=}{R}\overset{=\;}{T^{tr}}$$

(9) $$S_{n}^{gs}=e^{i\varphi_{n2}}\overset{=\;}{T^{t}}\overset{=}{R}\overset{=\;}{T_{n}^{r}}\overset{=\;}{F_{n}^{s}}\left(\pi -\theta_{i},\phi_{i};\theta_{r},\phi_{r},k_{i}\right)\overset{=\;}{T_{n}^{ir}}$$

(10) $$S_{n}^{gsg}=e^{i\varphi_{n3}}\overset{=\;}{T^{t}}\overset{=}{R}\overset{=\;}{T_{n}^{r}}\overset{=\;}{F_{n}^{s}}\left(\pi -\theta_{i},\phi_{i};\pi -\theta_{r},\phi_{r},k_{i}\right)\overset{=\;}{T_{n}^{rr}}\overset{=}{R}\overset{=\;}{T^{tr}}$$

(11) $$S_{m}^{dg}=e^{i\varphi_{n4}}\overset{=\;}{T_{m}^{i}}\overset{=\;}{F_{m}^{g}}\left(\theta_{i},\phi_{i};\theta_{r},\phi_{r},k_{i}\right)\overset{=\;}{T_{m}^{ir}}$$

where $\varphi_{1}$, $\varphi_{2}$, $\varphi_{3}$ and $\varphi_{4}$ are the time delay terms related to the first transmission paths as shown in Figure 1, and they can be obtained by $k_{0}\left[\left(\left|{\bar{r}}_{t}-{\bar{r}}_{n}\right|+\left|{\bar{r}}_{r}-\bar{r}{{}^{\prime }}_{n}\right|)-(\left|{\bar{r}}_{t}-{\bar{r}}_{n}\right|+\left|{\bar{r}}_{r}-{\bar{r}}_{n}\right|\right)\right]$,$k_{0}\left[\left(\left|{\bar{r}}_{t}-\bar{r}{{}^{\prime }}_{n}\right|+\left|{\bar{r}}_{r}-{\bar{r}}_{n}\right|\right)-(\left|{\bar{r}}_{t}-{\bar{r}}_{n}\right|+\left|{\bar{r}}_{r}-{\bar{r}}_{n}\right|)\right]$,$k_{0}\left[\left(\left|{\bar{r}}_{t}-\bar{r}{{}^{\prime }}_{n}\right|+\left|{\bar{r}}_{r}-\bar{r}{{}^{\prime }}_{n}\right|)-(\left|{\bar{r}}_{t}-{\bar{r}}_{n}\right|+\left|{\bar{r}}_{r}-{\bar{r}}_{n}\right|\right)\right]$ and $k_{0}\left[\left(\left|{\bar{r}}_{t}-{\bar{g}}_{n}\right|+\left|{\bar{r}}_{r}-{\bar{g}}_{n}\right|)-(\left|{\bar{r}}_{t}-{\bar{r}}_{n}\right|+\left|{\bar{r}}_{r}-{\bar{r}}_{n}\right|\right)\right]$, respectively.${\bar{r}}_{t}$ and ${\bar{r}}_{r}$ denote the coordinate vectors of the transmitter and receiver, and ${\bar{r}}_{n}$ and $\bar{r}{{}^{\prime }}_{n}$ denote the coordinate vectors of the scatterer and the mirror of the $n_{th}$ scatterer. $\bar{g}$ is the coordinate vector of the $m_{th}$ ground block. $\overset{=\;}{T_{n}^{m}}$ (*m* = *i*, *r* and *t*) denotes transmission matrix in incident direction, representing paths of top canopies-scatterer, ground-scatterer and top canopies-ground, respectively. $\overset{=\;}{T_{n}^{mr}}$ (*m* = *i*, *r* and *t*) denotes transmission matrix in scattering direction, representing paths of scatterer-top canopies, scatterer-ground and ground-top canopies, respectively. To obtain these transmission matrices, the vegetation area is divided into many small cubes and Foldy’s approximation [15] is used in the calculation process. $\overset{=}{R}$ is the specular reflection of ground surface. $\overset{=\;}{F_{n}^{s}}$ is the scattering matrix of the $n_{th}$ scatterer. $\overset{=\;}{F_{m}^{g}}$ is the scattering matrix of the $m_{th}$ ground block, and the amplitude is calculated by integral equation model [16], while the phase is set to be random. For both $\overset{=\;}{F_{n}^{s}}$ and $\overset{=\;}{F_{m}^{g}}$, $\left(\theta_{i},\phi_{i}\right)$ and $\left(\theta_{r},\phi_{r}\right)$ are the direction angles of transmitting and receiving antenna patterns. The generalized Rayleigh-Gans (GRG) approximation is used to calculate the scattering matrix from leaves. The method of infinite cylinder approximation and the generalized GRG approximation are used to calculate the scattering from trunk and branches [17]. The reflection matrix of ground surface is calculated by Fresnel reflection equation of plane.

### 2.2. UAV Robot Imaging Configuration Based on the Satellite Communication Systems

**Figure 2:**
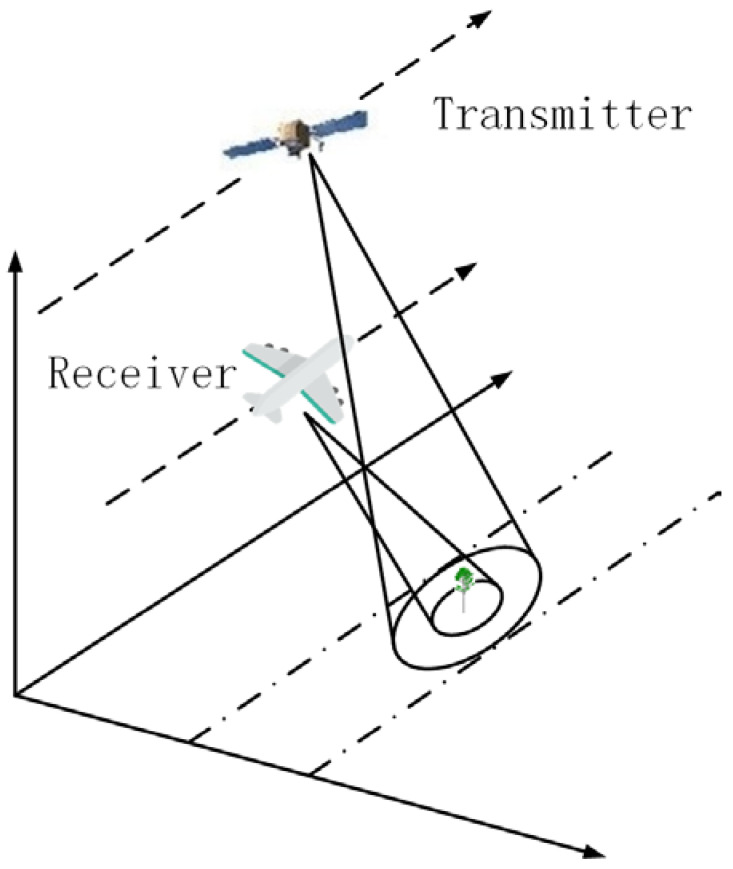
UAV robot observation configuration.

A UAV robot imaging system based on the satellite communication is one with the transmitter and receiver loaded on satellite and UAV platforms, respectively, as shown in Figure 2. In this simulation, the flight tracks of the transmitter and receiver are set to be parallel, and both transmitter and receiver operate in strip mode. Furthermore, it is assumed that both transmitter and receiver work in “work-stop-work” mode based on the fact that the motion velocity of this UAV and satellite is very small compared with the signal transmitting velocity. Therefore, the satellite can transmit and the UAV robot can receive the signals at a series of discrete positions. In this study, the orbit altitude of the transmitter remains unchanged, while the heights of the receivers loaded on UAV networking are considered to obtain scattering signals at different zenith angles, which contribute to analyze the multi-path scattering mechanisms of vegetation based on UAV networking using a satellite communication system.

The working frequency is chosen at C band, and proper bandwidth is selected according to the system, as listed in Table 1. The altitude of the transmitter is 800 km, while the height of receiver is 8 km. The incident angle of the transmitting signal is $45^{\circ }$, and it can be demonstrated that the horizontal distance between the transmitter and the target vegtation is approximately 800 km. For the receiver, six typical zenith angles: $26^{\circ }$, $36^{\circ }$, $46^{\circ }$, $56^{\circ }$, $66^{\circ }$ and $76^{\circ }$ are chosen to study how the zenith angle affects the multi-path scattering imaging results. The horizontal distance between the receiver and the target vegetatioin can be calculated according to the zenith angle and flight height. Other parameters in this simulation are listed in Table 1.

## 3. Imaging Method

In this study, the passive imaging of the UAV robot using the satellite communication systems is developed from the field imaging algorithm [18]. The basic idea of this algorithm is to obtain the target equation from the measurement field through the inverse Fourier transform. The target equation is a function of the target position. To realize the target imaging, the scattering fields from multiple observing angles and frequencies need to be measured.

In this study, both transmitter and receiver work in “work-stop-work” mode which is applied to observe the vegetation scene as shown in Figure 2, and the field imaging algorithm suitable for this observing system is adopted to process the data. The UAV and satellite move in two straight lines parallel to the edge of the target area, respectively. The received signal of the UAV robot can be expressed as

(12) $$E\left(\bar{k}\right)=\sum_{n=1}^{N}e^{i\bar{k}\cdot {\vec{r}}_{n}}F_{n}\left(\bar{k}\right),$$

where $\bar{k}={\bar{k}}_{i}-{\bar{k}}_{s}$. ${\bar{k}}_{i}$ and ${\bar{k}}_{s}$ are the wave vectors of incident and scattered wave. *N* denotes the total number of scatterers. $F_{n}\left(\bar{k}\right)$ is the scattering amplitude of the nth scatterer. It is known that the received signal is an equation about the wave number and the receiving antenna position. Here, the orbit altitude of the transmitter of the transmitting radar remains unchanged.

**Figure 3:**
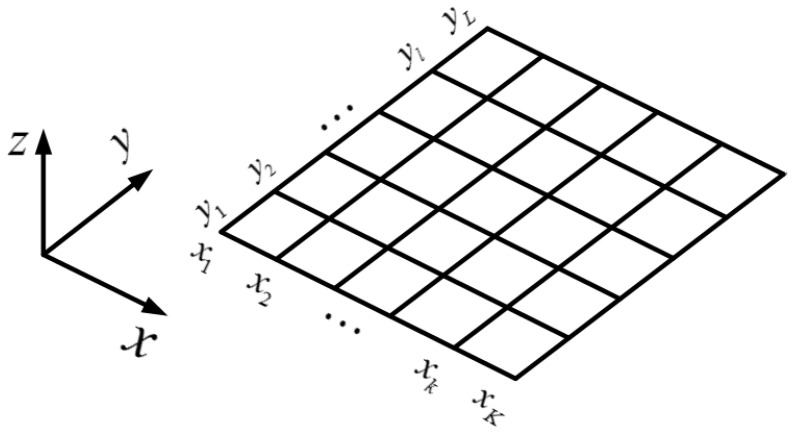
The grid division of imaging scene.

In the actual calculation of the target function $F_{n}\left(\bar{k}\right)$, the received signal $E\left(\bar{k}\right)$ is not directly transformed by the inverse Fourier transform but by the conjugate multiplication and integration of received signal and the reference signal. The target of this study is virtual vegetation. The echo signal comes from the coherent scattering model of vegetation. The field imaging algorithm is to conjugate and multiply the echo signal at frequency $k_{m}$ and position $x_{n}$ with the reference signal, and then add and sum the echo signal at all frequencies and receiving antenna positions. Furthermore, the observation area is divided into *K* × *L* pixel units according to the requirement of imaging equality as shown in Figure 3. Each unit has a pixel center, which can be employed for the coordinate position of reference phase. The scattering intensity of pixel unit at ${\bar{r}}_{0}$ can be expressed as

(13) $$E_{est}^{s}({\bar{r}}_{0})=\frac{1}{N_{k}N_{x}}\sum_{m=1}^{N_{k}}\sum_{n=1}^{N_{x}}E(k_{m},x_{n}^{t},x_{n}^{r})E_{0}{\left(k_{m}\right)}^{*}e^{-ik_{m}\left(\left|{\bar{R}}_{t}(x_{n}^{t})-{\bar{r}}_{0}\right|+\left|{\bar{R}}_{r}(x_{n}^{r})-{\bar{r}}_{0}\right|\right)},$$

where $N_{x}$ and $N_{k}$ represent the number of spatial sampling points of receiving antenna and discrete frequency points, respectively. The variable ${\bar{R}}_{t}(x_{n}^{t})=x_{n}^{t}\hat{x}+y_{n}^{t}\hat{y}+z_{n}^{t}\hat{z}$ and ${\bar{R}}_{r}(x_{n}^{r})=x_{n}^{r}\hat{x}+y_{n}^{r}\hat{y}+z_{n}^{r}\hat{z}$ represent the position vectors of transmitting and receiving antennas in the global coordinates. The position of the pixel center (*k, l*) is ${\bar{r}}_{0}=x_{k}\hat{x}+y_{l}\hat{y}+0\hat{z}$ in the global coordinates. $E_{0}(k_{m})$ is the reference signal. $E(k_{m},x_{n}^{t},x_{n}^{r})$ can be calculated by Equation (1). If we chose the different position ${\bar{r}}_{0}$ in Figure 3, Equation (13) can then be mapped into the imaging plane to reconstruct the multi-path scattering map.

## 4. Results

In this simulation, we assume that the transmitter moves from −50 km to 50 km in the azimuthal direction, and that the receiver moves from −500 m to 500 m in the azimuthal direction. The frequency of signal gathering is from 6 GHz to 6.3 GHz with a step of 6 MHz. In the following, the visual vegetation is introduced firstly, and then the passive imaging results using multi-path scattering from single tree and multiple trees are presented.

### 4.1. Virtual Vegetation

In this study, L-system [19,20] is used to generate the 3-D spatial structures of trees, and the structure parameters of the tree are shown in Table 2. The dielectric constants of the tree elements and the ground surface at C band used in this simulation model are listed in Table 3.

The ground surface is divided into many blocks according to the cell size used in calculating the canopy attenuations. The side length of the ground surface is 1 m. Moreover, the *rms* height and correlation length of the ground surface are set to be 1.5 cm and 15 cm, respectively.

### 4.2. The Passive Imaging Results Using Multi-Path Scattering from a Single Tree

**Figure 4:**
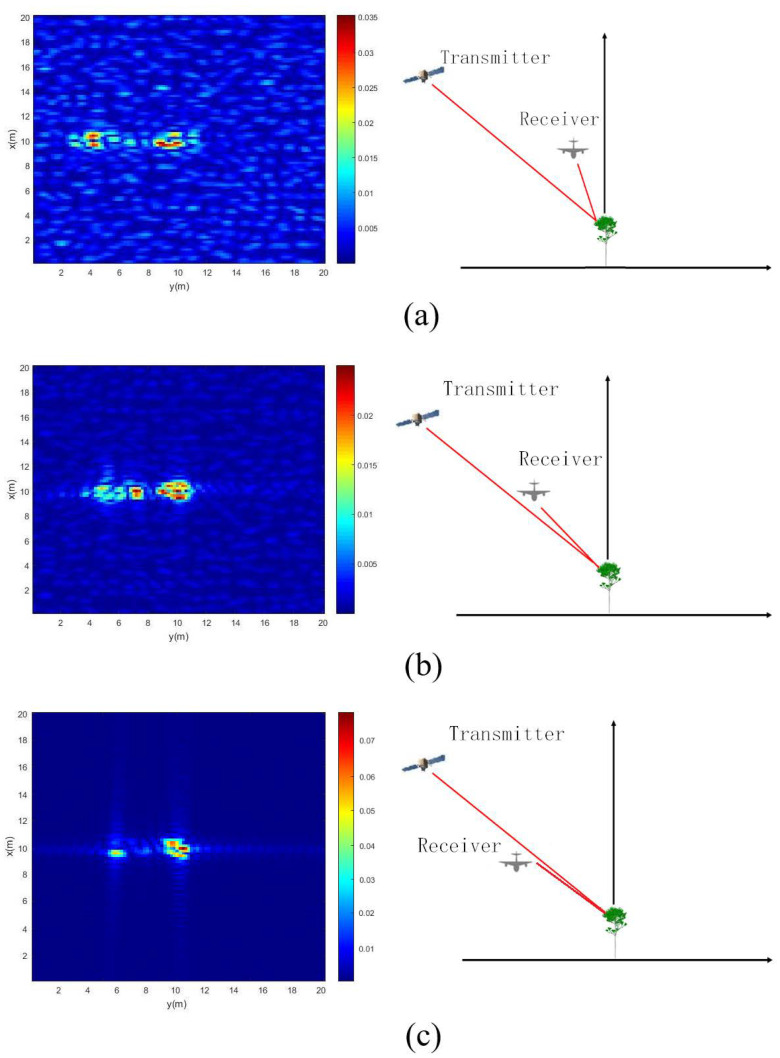

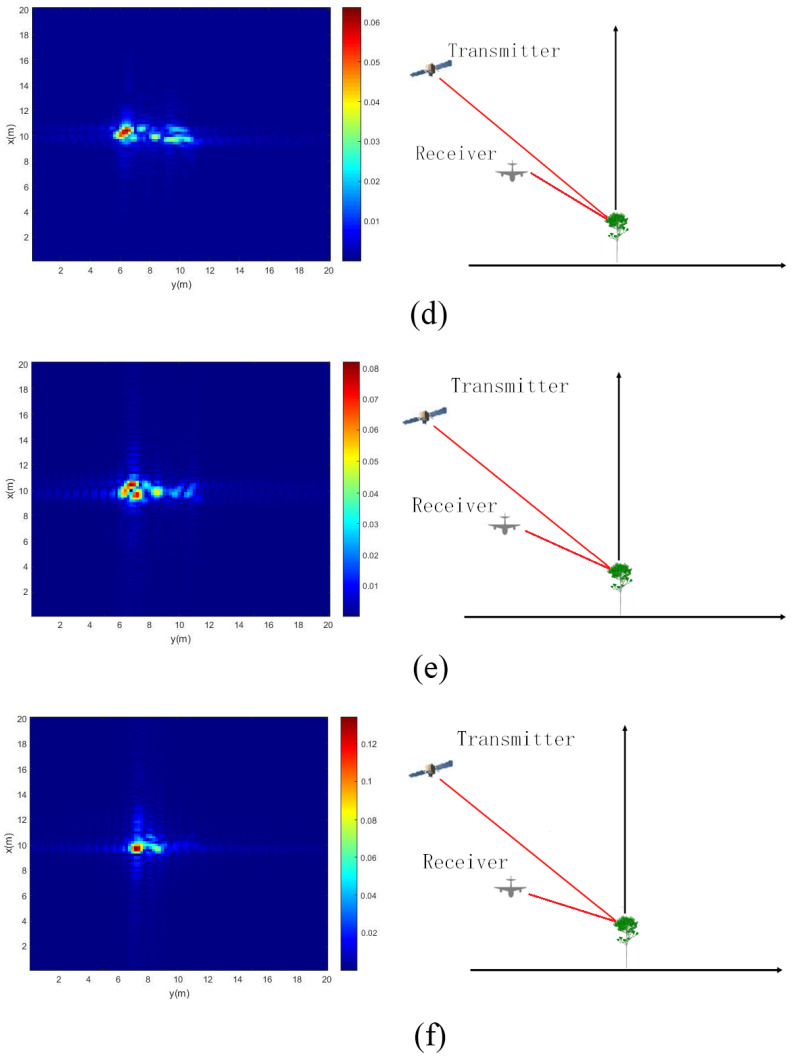
UAV imaging results of a single tree at different receiver zenith angles: (**a**) receiving zenith angle = $26^{\circ }$; (**b**) receiving zenith angle = $36^{\circ }$; (**c**) receiving zenith angle = $46^{\circ }$; (**d**) receiving zenith angle = $56^{\circ }$; (**e**) receiving zenith angle = $66^{\circ }$; (**f**) receiving zenith angle = $76^{\circ }$.

Firstly, the simulation results of a single 5 m high tree located at the position (10 m, 10 m) in the coordinate system is presented and discussed. The height of the flight track of the transmitter and receiver remains unchanged, while the position of the receiver is changed to be at six different zenith angles. The model presented in Section 2 is used to simulate the electric field of this tree at different frequency points from 6 GHz to 6.3 GHz, with a step of 6 MHz and a position from −500 m to 500 m with a step of 5 m. Next, the simulating electric fields received by UAV robot are processed by the imaging method described in Section 3 to obtain the images. The following six images are obtained by above methods at zenith angles of $26^{\circ }$, $36^{\circ }$, $46^{\circ }$, $56^{\circ }$, $66^{\circ }$ and $76^{\circ }$, respectively, as shown in Figure 4a–f. The scene size is 20 m × 20 m, and the size of each pixel is 0.2 m × 0.2 m.

From the images, it can be seen that the simulation results demonstrate good performance of the autofocus based on the passive imaging algorithm using multi-path scattering. The energy concentrates on two main parts; the left part is mainly from the direct scattering of the scatterer in the vegetation, and the right part is the summation of a single bounce between the scatterer and the ground. The double bounce of ground-scatterer-ground is too weak to be shown in the images.

### 4.3. The Passive Imaging Results Using Multi-Path Scattering from Multiple Trees

**Figure 5:**
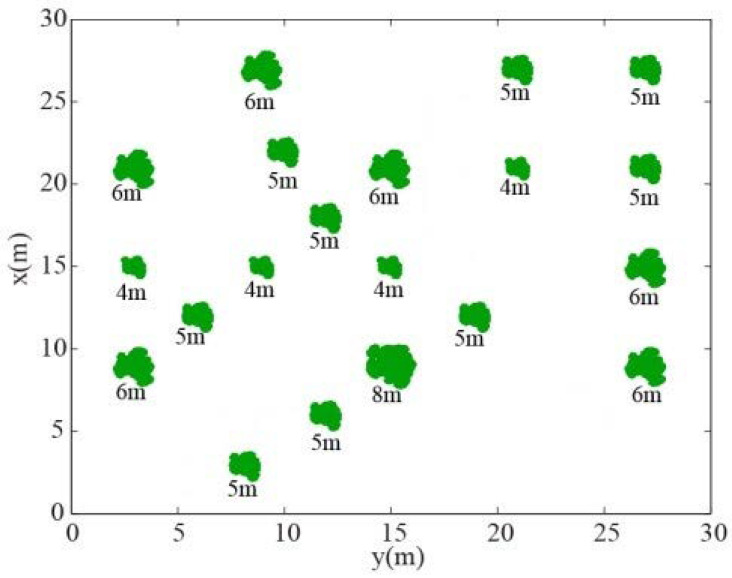
The distribution and height of each tree in a stand.

Furthermore, multiple trees in an area of 30 m × 30 m are generated by the L-system. The distributions and heights of trees in this area are shown in Figure 5.

**Figure 6:**
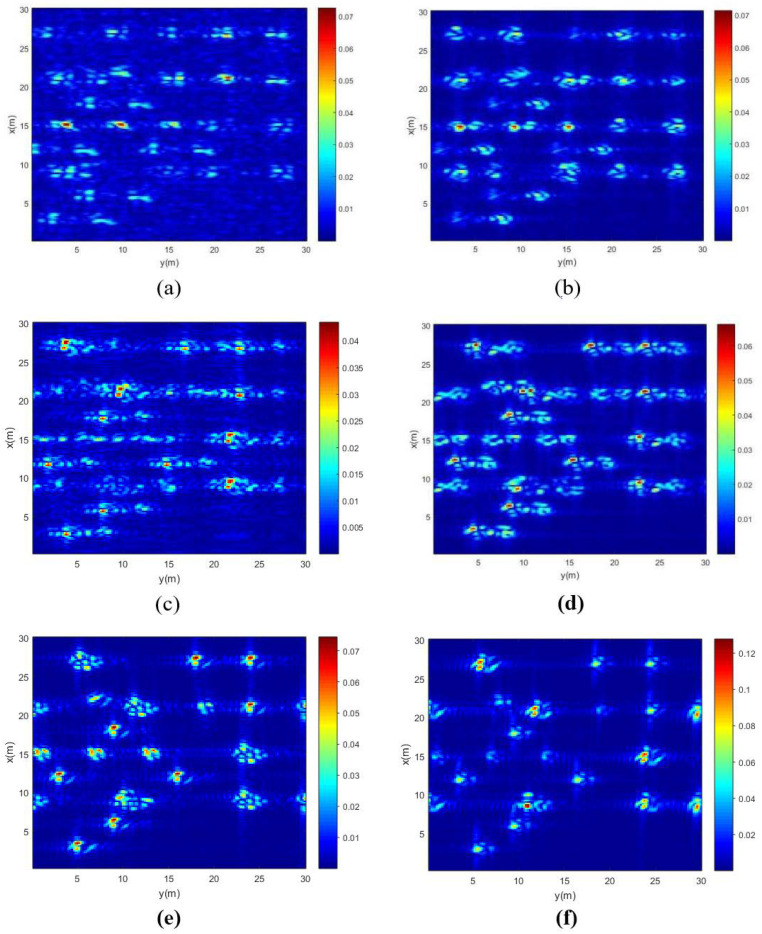
UAV imaging results of trees at different receiver zenith angles: (**a**) receiving zenith angle = $26^{\circ }$; (**b**) receiving zenith angle = $36^{\circ }$; (**c**) receiving zenith angle = $46^{\circ }$; (**d**) receiving zenith angle = $56^{\circ }$; (**e**) receiving zenith angle = $66^{\circ }$; (**f**) receiving zenith angle = $76^{\circ }$.

Figure 6 shows the bistatic UAV microwave imaging results of multiple of trees in this stand. The height of flight track of the transmitter and receiver also remains unchanged, while the position of the receiver is changed to be at six different zenith angles. The rest of the bistatic UAV microwave imaging parameters are the same as those in Section 2.2. Six imaging results of multiple trees are obtained at zenith angles of $26^{\circ }$, $36^{\circ }$, $46^{\circ }$, $56^{\circ }$, $66^{\circ }$ and $76^{\circ }$, respectively, as shown in Figure 6a–f.

## 5. Discussion

From above simulation results, based on the imaging method proposed in this study, it can be seen that the larger the receiver’s zenith angle is, the closer the distance between two parts of energy becomes. This is because the distributions of scattering energy in these images are related to the travel path and the imaging algorithm. When the receiver’s zenith angle is larger, the lengths of propagation paths from direct scattering and single bounce become closer. For this imaging configuration, it can be inferred that when the receiver’s zenith angle becomes large enough, the two parts will overlap.

**Figure 7:**
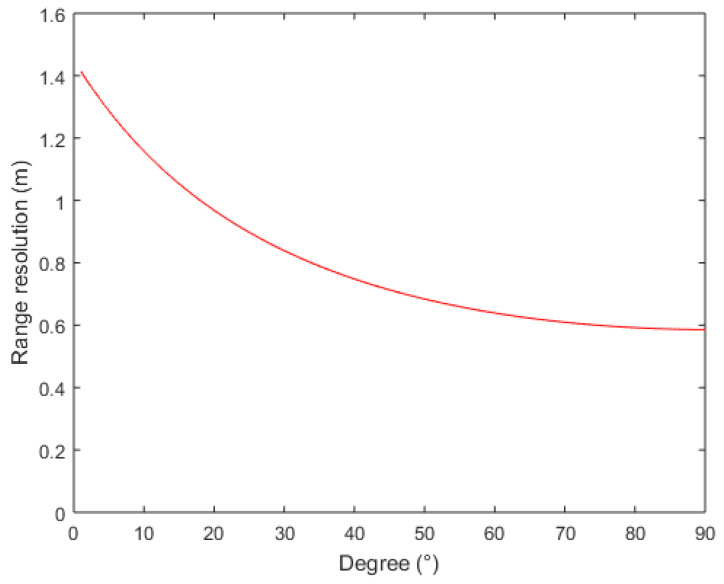
The range resolutions as a function of the zenith angle of a receiver.

In addition, it can be observed that the range resolution of Figure 6c–f is relatively clear among the six images. The resolution of the image shown in Figure 6b is somewhat worse than that in Figure 6c, but better than that in Figure 6a. These results might be due to the variation in the receiver’s zenith angle. Equation (14) presents the range resolution formula [21], where *c* is the velocity of an electromagnetic wave propagating in free space, and *B* is bandwidth. $\theta_{t}$ and $\theta_{r}$ are the zenith angles of transmitter and receiver, respectively. The range resolutions as a function of zenith angle of receiver are calculated based on Equation (14), as shown in Figure 7. The range resolutions of six zenith angles of receivers shown in Figure 6 are listed in Table 4. The calculation results contribute to the understanding of the aforementioned imaging results.

(14) $$\Delta L_{range}=\frac{c}{B(\sin(\theta_{t})+\sin(\theta_{r}))}$$

Furthermore, from Figure 6, it is noticed that the zenith angle of the receiver also affects the signal strength received from the ground surface. Direct scattering from the ground surface is clearly shown in Figure 6a. However, as the zenith angle of the receiver increases, the signal strength from the ground surface becomes weaker and weaker. For Figure 6c, the signal strength received from the ground surface is too weak to be distinguished. In addition, it can be seen from the figures that the scattering strength of a single tree from the left part is gradually stronger than that on the right part. We have suggested in Section 4.2 that the image of a single tree includes two main parts: the left part is mainly from direct scattering of the scatterer in the tree, and the right part is the summation of a single bounce between the scatterer and ground. For a large receiving angle, the signal strength received from the ground surface is relatively weak. Furthermore, the strength from right part in the figure will also weaken because the right part depends on a single bounce between the scatterer and the ground. Therefore, it can be seen that the signal strength from the left part becomes gradually stronger, as shown in Figure 6a–f.

For imaging results of multiple trees in a stand as shown in Figure 6, it can be found that when the receiver zenith angle is large, the distribution of trees in this area is relatively clear, and different scattering components of a single tree are relatively close. When the receiver zenith angle is small, the scattering component received from the ground surface is strong. To a certain extent, some scattering components of the tree are suppressed by direct scattering components of the ground surface, as shown in Figure 6a. In Figure 6a, we cannot clearly distinguish the complete scattering information of every tree, we can only roughly distinguish the position of every tree from the image.

## 6. Conclusions

This article presents the simulation results of UAV robot passive imaging using the satellite communication systems based on the multi-path scattering model. In this UAV robot imaging configuration, the incident angle of the transmitter remains unchanged, while the different flight tracks of receivers loaded on different UAVs result in different zenith angles. Furthermore, the simulated signals received from the multi-path scattering model at each position are processed by a passive imaging algorithm. From the imaging results of vegetation, we can see that different zenith angles of the receiver affect the imaging results. When the receiving and transmitting antenna is located at the same side of the target area, the greater the zenith angle of the receiving antenna is, the higher the range resolution of the UAV robot passive imaging is. The combination of multi-path scattering model and the imaging algorithm provides an effective method to study the scattering mechanism based on vegetation. At the same time, the method can be utilized to study the effects of vegetation scattering. The simulation results offer a reference for the development and research of UAV robot passive imaging using the satellite communication systems in the field of ground microwave monitoring.

## Figures and Tables

**Table 1:** Parameters of the observation geometry.

Parameters	Value
Start frequency	6 GHz
Frequency bandwidth	300 MHz
Frequency sampling numbers	50
Flight height of the transmitter	800 km
Flight height of the receiver	8 km
Space sampling interval of transmitter	500 m
Space sampling interval of receiver	5 m
Space sampling numbers	201
Polarization	HH

**Table 2:** Structure parameters of trees.

Height of Trees		4 m	5 m	6 m
Leaf	Radius (m)	0.04	0.04	0.04
	Thickness (mm)	0.15	0.15	0.15
	Density (N m^−3^)	1360.0	580.0	250.0
Branch	Range of radius (cm)	0.1–1.6	0.1–2.2	0.1–2.7
	Range of length (cm)	1.0–103.8	1.0–178.8	1.0–195.0
	Density (N m^−3^)	180.0	60.0	45.0
Crown	Height (m)	2.0	3.0	3.2
	Width (m)	1.2	1.5	2.2

**Table 3:** The dielectric parameters of scatterers.

Parameters	Value
Leaves	20.24 + i6.78
Trunks	12.30 + i4.16
Branches	12.30 + i4.16
Ground	9.6 + i2.04

**Table 4:** Range resolution of different receiving angles.

Zenith Angle of Receiver (°)	Value (m)
26	0.87
36	0.77
46	0.70
56	0.65
66	0.62
76	0.60

## Data Availability

Data available on request from the authors.
